# Socio-economic health inequalities through the lens of social justice theory, an innovative perspective

**DOI:** 10.1016/j.puhip.2025.100630

**Published:** 2025-06-25

**Authors:** Jantien van Berkel, Michèlle Bal

**Affiliations:** Utrecht University, Faculty of Social and Behavioral Sciences, Department of Interdisciplinary Social Science, Chair Group Public Health, P.O. box 80140, 3508 TC, Utrecht, the Netherlands

**Keywords:** Health inequalities, Health promotion, Inequalities

## Abstract

**Background:**

Socioeconomic health inequalities, characterized by avoidable, systematic, and unfair differences in health between social groups based on socioeconomic disparities, persist in Western countries despite extensive efforts. This situation prompts a critical examination of current public health interventions. While individual-focused approaches emphasize behavioral strategies to address avoidable factors, and systems approaches target systematic causes, there remains a significant gap in addressing one crucial aspect of health inequalities: their inherent unfairness.

**Study design:**

In this contribution, we conduct a theoretical exemplification.

**Methods:**

Distributive, procedural, and recognitive justice theory has been employed as critical lens to analyze public health interventions and policies.

**Results:**

Distributive justice focuses on the fairness of outcomes and is recognizable in the (re)distribution of resources such as food aid. However, if redistribution comes with stigma attached, it still fails to address fairness in full, which can hamper uptake. Procedural justice focuses on the fairness of the process leading up to certain outcomes. Stakeholder engagement in policy development is an example, but avoiding tokenism is key for truly reducing unfairness. Finally, recognitive justice emphasizes recognition, human dignity and equal social participation. It might be most elusive to ‘catch’ in policy but is also most crucial to address unfairness completely.

**Conclusions:**

Integrating social justice theory into public health strategies promotes fairness and contributes to of the reduction of health inequalities.

## 1 Introduction

Socioeconomic health inequalities persist in Western countries, showing significant differences in life expectancy and health between advantaged and disadvantaged groups. Defined as “systematic, avoidable, and unfair differences in health outcomes observed between populations, social groups, or across a population ranked by social position” [1], these inequalities have garnered substantial attention in public health discourse. Despite efforts, health inequalities persist and have even worsened in some countries like the Netherlands [2]. This raises the question: Are current public health approaches addressing the root causes?

Traditionally, public health has focused on improving population health by preventing diseases. In recent decades, public health has undergone a notable shift, often referred to as the “behavioral turn,” with a predominant focus on individual behavior change and health promotion through targeting *avoidable* differences between social groups [3]. Lifestyle factors like physical activity, smoking, diet, and stress became primary intervention targets due to their link to life expectancy and health inequality. However, this emphasis on individual behavior faced criticism for its narrow focus, often overlooking broader social determinants of health and perpetuating what has been termed “lifestyle drift” [4]. Despite considerable investment and effort, meta-analyses have consistently shown limited to no effects of behavioral interventions in reducing health inequalities. Thus, while lifestyle factors are undeniably linked to health inequality, interventions targeting individual behavior have failed to meaningfully address the underlying systematic causes of these inequalities.

Recognizing the limitations of individual-focused approaches, public health is increasingly turning towards so-called systems-approaches to address the complexity inherent in health inequalities [5]. These systems-approaches acknowledge the intricate interplay between individual behaviors and broader socio-environmental factors, emphasizing upstream Social Determinants of Health [6] and the systematic nature of inequalities [3]. However, while this “systems turn” offers promise in addressing the structural roots of health disparities, both the behavioral and systems approaches often fall short in explicitly addressing the inherent unfairness embedded within health inequalities. However, while this “systems turn” offers promise in addressing the structural roots of health disparities, both behavioral and systems approaches often overlook the deeper question of what makes some health inequalities unfair—and how to address them fairly.

This oversight underscores the need for a more comprehensive theoretical grounding of (addressing) fairness in health inequalities. Drawing on established strands of social justice theory, this paper offers a theory-driven perspective to better understand and address the unfairness underlying socio-economic health inequalities.

## 2 Methods

This short communication presents a theory-informed application of social justice theory applied to interventions and policies addressing health inequalities.

## 3 Results

### 3.1 Distributive justice

Distributive justice interventions focus on (re)distributing resources (and alleviating burdens for targeted groups) to achieve more fair outcomes [7]. This (re)distribution occurs when a lack of certain resources results in perceived unfairness, prompting interventions to address differences. Consider the example of a “sport fund” implemented by municipalities to address disparities in sports participation among children from low-income (vs. high-income) families. Due to financial constraints, these children often lack access to sports activities, which can impact their physical and mental well-being. This lack of access can be considered unfair as you have no influence over where you are born. Through the establishment of a dedicated fund, families with limited economic resources can apply for financial assistance to cover membership fees, sports equipment, and other related expenses. By this (re)distribution of resources, this intervention enables more equitable access to sports opportunities, thereby promoting more fair outcomes among children from diverse socioeconomic backgrounds. However, when there is stigma attached to the use of these policies (i.e., recognitive injustice), their uptake will likely fall behind [8].

### 3.2 Procedural justice

Procedural justice focusses on fairness of the processes leading up to certain outcomes [9]. Within interventions and policies, procedural justice oftentimes centers on stakeholder engagement, particularly emphasizing the inclusion of those directly affected by the issue at hand but typically excluded from decision-making processes. Incorporating individuals with lived experiences, often referred to as experts-by-experience, in the development or selection of interventions is widely regarded as fair. However, ensuring a fair process and reaping its benefits extends beyond merely “offering a seat at the table”. The positive effects of providing voice necessitate genuine inclusion (i.e., recognitive justice), where stakeholders' voices are not only present but carefully listened to and considered. This underscores the importance of avoiding tokenism (i.e. making only a symbolic effort to include members of underrepresented groups) and instead fostering meaningful involvement, wherein decision-making power is truly shared among stakeholders.

### 3.3 Recognitive justice

Recognitive justice refers to the dimension of fairness that emphasizes the importance of acknowledging and valuing individuals and social groups in decision-making processes, highlighting the need for genuine consideration, beyond mere (re)distribution of resources and procedural fairness [7]. Integrating recognitive justice into interventions and policies is crucial for ensuring that marginalized social groups feel genuinely acknowledged and respected in their identities and experiences. Based on Honneth's framework [10], we identify three key forms of social recognition: need-based care, equality-based respect, and achievement-based social esteem. Need-based care involves recognizing and addressing individual needs, which provides emotional stability and a sense of personal value. This is exemplified in personalized care approaches, where the emphasis is on listening and responding to individual needs rather than making assumptions—highlighting the significance of tailored care.

Equality-based respect is rooted in the recognition of equal rights, which has evolved from a primarily legal concept—such as the rights for same-sex marriage or the recognition of diverse identities through options like an “X" on passports—into a broader social and cultural practice. This evolution is reflected in the shift toward inclusive policy language, such as using “people-first” terminology (e.g., “families in multi-problem situations” rather than “multi-problem families”) and moving away from hierarchical distinctions like “higher” and “lower” educated.

Lastly, achievement-based social esteem, in contrast, is closely tied to individual accomplishments, particularly within professional contexts, reflecting a capitalist societal framework where work often defines one's worth. This focus on professional achievement, however, can marginalize those without employment, leading to a lack of recognition and potential social and psychological repercussions. An example of an intervention addressing this issue is stress-sensitive debt counseling, which (amongst other things) draws on humanistic psychology to offer unconditional positive regard, acknowledging individual efforts and fostering esteem in the challenging circumstances of debt.

While social recognition can be considered crucial to address health inequalities, it is important to acknowledge that it must work in tandem with distributive and procedural justice to achieve fair interventions and policies. For instance, an asset-based approach to reintegration for unemployed individuals, which emphasizes the discovery and utilization of their talents, can enhance social recognition by valuing these aspects. However, if this approach is implemented without addressing the underlying socioeconomic conditions (requiring a distributive justice approach) and/or without involving the individuals in decision-making processes (a key aspect of procedural justice), it risks perpetuating rather than alleviating injustice. Therefore, for interventions to be genuinely fair and effective, they must integrate all three dimensions of justice: recognitive, distributive, and procedural.

Table 1 demonstrates how applying the three strands of social justice theory can surface hidden assumptions and limitations in conventional public health interventions. While many interventions aim to promote equal outcomes (distributive justice), they may fall short in ensuring inclusive processes (procedural justice) or affirming social identities and lived experiences (recognitive justice). A justice-oriented lens can thus both broaden and deepen our understanding of what makes an intervention truly fair.

**Table 1:** Illustrative analysis of conventional public health interventions through the lens of social justice theory.

Public Health Intervention	Distributive Justice (Fair distribution of outcomes and resources)	Procedural Justice (Fairness in decision-making processes)	Recognitive Justice (Recognition of identities, experiences, and contributions)
School breakfast programs	Aims to reduce nutritional inequalities by offering free meals to all students, especially benefiting those from low-income households.	Often implemented top-down, with limited participation of families in design (e.g. what foods are offered, at what times, and how). Procedural justice would include co-design with parents, school staff and students, acknowledging lived experiences of food insecurity.	Recognizes children's needs but may unintentionally stigmatize those who use the program (both children – especially when separated from others - and parents). Moreover, universal programs may fail to address unequal needs (e.g. children with food allergies or specific cultural diets). Recognitive justice requires universal access without labelling, and sensitivity to cultural and familial values around food.
Smoking cessation campaigns	Focuses on behaviour change and reducing tobacco-related health risks, often using mass media or subsidized cessation tools. Interventions typically designed to reach lower income smokers yet may not sufficiently account for broader structural determinants (e.g. financial stress).	Usually designed by experts, with little involvement from target groups in shaping messages or delivery. Procedural justice would involve giving decision-making power to community members, ensuring relevance and legitimacy.	Risk of moralizing or stigmatizing smokers—especially low-income groups—by framing smoking as individual failure. Recognitive justice implies equality-based respect, avoiding shaming language and acknowledging the crucial need to (also) target underlying circumstances (e.g. financial stress).
Digital health apps (e.g. for exercise, food intake, mental health)	May expand access to health information and behavior change, but assumes digital literacy and access to technology, which are distributed unequally, thereby risking increasing inequalities.	Often designed by experts, although involvement of intended users (in testing) is becoming more common. Procedural justice would not only require participatory design from the start with diverse user groups, but also transparency in data governance.	Assuming a universal user profile may overlook diverse identities. For recognitive justice, designs should not only offer inclusive visuals and language, but also a full-fledged offline alternative to acknowledge the full range of capacities (including no access).

## 4 Discussion

In this short communication, we apply social justice theory explicitly to public health interventions and policies, to address the inherent unfairness of socioeconomic health inequalities. While unfairness is a fundamental aspect of the definition of health inequality, literature combining health inequality and social justice remains relatively sparse. Therefore, our discussion of the combination of multiple dimensions of social justice, particularly distributive, procedural, and recognitive justice, and their applicability in interventions and policies represents an innovative contribution to the field.

We highlight the difference between fairness and equality in addressing health inequalities. Interventions should not only distribute resources according to a fair distributive justice principle (e.g., equality) but also respect dignity and autonomy (recognitive justice). For instance, offering healthy food at food banks may decrease inequality and address distributive justice but perpetuate stigma, undermining recognitive justice. Strategies such as making healthy food more affordable or adopting consumer-based food aid approaches may better uphold both distributive and recognitive justice principles. When arrived at through a fair decision-making process (procedural justice), it holds potential to effectively contribute to reducing health inequalities. The combination of all three dimensions of social justice is thus pivotal to address the unfairness of health inequalities.

However, it is important to acknowledge the challenges of applying social justice theory in addressing health inequalities. Social justice is a contested concept, with varying interpretations and political implications. Moreover, power dynamics and system justifying tendencies can influence these interpretations. While we advocate for the inclusion of social justice considerations in health policies and interventions, we recognize the difficulty associated with achieving consensus on its conceptualization and operationalization. Our intention here is not to advocate for radical societal transformation but rather to integrate social justice principles as we have outlined them here into the design, implementation and evaluation of interventions and policies.

We acknowledge that our paper may not encompass all aspects of social justice, as it represents one approach among many. Nevertheless, by explicitly integrating principles of procedural, distributive, and recognitive justice, we propose that interventions and policies more effectively target fairness in addressing health inequalities. Ultimately, embracing social justice as guiding principles helps challenge entrenched inequalities, and strive towards more fair and inclusive societies.

## What this study adds

• **A theory-driven perspective on health inequalities:** This study draws on established strands of social justice theory — distributive, procedural, and recognitive justice — not as descriptive labels, but as theorized perspectives that deepen current understandings of the fairness of socio-economic health inequalities.
• **Bridging theory and practice:** By applying theoretical insights to practical public health interventions, the study provides actionable recommendations aimed at promoting fairness in health outcomes.
• **A contribution to public health innovation:** The study advances the field by demonstrating how a justice-oriented approach can inform the design and implementation of effective strategies to tackle socio-economic health inequalities.

## Implications for policy and practice

• **Informing policy design and interventions:** Integrating social justice principles into health policies and interventions can address the unfairness of socio-economic health inequalities.

## Ethical approval

This study did not need ethical approval, as it is not empirical, and did not involve any human subjects.

## Funding

This study received no funding.

## Competing interests

The authors declare they have no competing interests.
